# Cadherin-11 cooperates with inflammatory factors to promote the migration and invasion of fibroblast-like synoviocytes in pigmented villonodular synovitis

**DOI:** 10.7150/thno.48666

**Published:** 2020-08-21

**Authors:** Chenxi Cao, Fei Wu, Xingyue Niu, Xiaoqing Hu, Jin Cheng, Yan Zhang, Chuanyun Li, Xiaoning Duan, Xin Fu, Jiying Zhang, Xin Zhang, Yingfang Ao

**Affiliations:** 1 Institute of Sports Medicine, Beijing Key Laboratory of Sports Injuries, Peking University Third Hospital, 49 North Garden Road, Haidian District, Beijing 100191, People's Republic of China.; 2 State Key Laboratory of Membrane Biology, Institute of Molecular Medicine, Peking University, 5 Yiheyuan Road, Haidian District, Beijing 100871, People's Republic of China.; 3 Institute of Molecular Medicine, Beijing Key Laboratory of Cardiometabolic Molecular Medicine, Peking University, 5 Yiheyuan Road, Haidian District, Beijing 100871, People's Republic of China.

**Keywords:** pigmented villonodular synovitis, cadherin-11, inflammation, fibroblast-like synoviocytes, targeted therapy

## Abstract

**Rationale:** Pigmented villonodular synovitis (PVNS) is a destructive benign tumor-like hyperplastic disease that occurs in synovial tissue. Fibroblast-like synoviocytes (FLS) are the predominant cell type comprising the structure of the PVNS synovial lining layer. Due to a high recurrence rate, high invasion, migration, and cartilage destruction ability, PVNS causes substantial damage to patients and the efficacy of surgical resection is not satisfactory. Therefore, exploring the pathogenesis and identifying novel therapeutic targets for PVNS are urgently required. Currently, the pathogenesis of PVNS remains unclear, and there is uncertainty and controversy regarding whether PVNS is an inflammatory or a neoplastic disease. Cadherin-11 is a classical molecule that mediates hemophilic cell-to-cell adhesion in FLS and plays an important role in the normal synovium lining layer formation. This study aimed to explore the role of inflammation and cadherin-11 in PVNS pathogenesis and determine the effects of cadherin-11 as a molecular target for PVNS treatment.

**Methods:** FLS were primarily cultured from PVNS patients during arthroscopic synovectomy. The level of cytokines in the PVNS synovial fluid was evaluated using a human antibody array. Cadherin-11 expression of PVNS FLS was detected by qPCR, Western blots, tissue immunohistochemistry, and cell immunofluorescence. Cadherin-11 was down-regulated by siRNA or up-regulated with a plasmid, with or without inflammatory factor stimulation, and PI3K/Akt was inhibited with LY294002. The capacity of migration and invasion of PVNS FLS was tested using Transwell and wound-healing assays. Activation of the nuclear factor-kappaB (NF-κB) and mitogen-activated protein kinase (MAPK) pathways was detected by Western blots. Chondrocyte damage by PVNS FLS was assessed with a co-culture assay.

**Results:** Inflammatory factors (IL-1β and TNF-α) in the synovial fluid of PVNS patients were significantly up-regulated. Cadherin-11 was highly expressed in the FLS of PVNS patients, and positively correlated with recurrence, extra-articular migration, and cartilage destruction of PVNS. Knocking down of cadherin-11 inhibited the migration and invasion of PVNS FLS. Moreover, inflammatory factors up-regulated the expression of cadherin-11, which activated the NF-κB and MAPK signaling pathways and led to cartilage destruction. Inhibition of cadherin-11 blocked IL-1β- and TNF-α-induced activation of the above pathways, migration and invasion of PVNS FLS, and damage of chondrocyte. In addition, the elevation of cadherin-11 expression, together with the migration and invasion, of PVNS FLS was down-regulated by the inhibition of the PI3K/Akt signaling pathway.

**Conclusions:** Cadherin-11 plays an important role in the pathogenesis of PVNS and forms a positive feedback loop with inflammatory factors, which further activates the NF-κB and MAPK pathways to trigger an inflammatory cascade. Cadherin-11-mediated inflammation results in PVNS with high recurrence, invasiveness, and strong cartilage destruction ability, and eventually promotes the transformation of PVNS from the initial inflammatory disease to neoplastic disease. Thus, inhibition of cadherin-11 together with its related inflammatory reaction, represents a new therapeutic strategy for PVNS.

## Introduction

Pigmented villonodular synovitis (PVNS), also known as a diffuse-type tenosynovial giant cell tumor (TGCT), is a benign neoplasm that is often a locally aggressive process arising from the intra-articular or tenosynovial tissue of an uncertain etiology 1. The estimated incidence of PVNS is 1.8 per million individuals and it has a high rate of local recurrence, especially the diffuse type 2. Due to the atypical clinical symptoms of PVNS, the diagnosis is often delayed and most patients present with slowly progressive monoarticular disease, exhibiting joint bleeding, swelling, and long-term pain in the affected joints that leads to a functional impairment and osteoarticular destruction 3. In addition, previous studies have reported that PVNS undergoes malignant transformation and causes metastasis in other parts of the body 4. It is still controversial whether PVNS is an inflammatory or a neoplastic disease 5, 6. Since its pathogenesis is currently unclear, the therapeutic mainstay of PVNS is limited to removing the entire pathological synovial tissue to relieve pain and lower the risk of joint destruction, whereas local recurrence is typically elevated after a simple synovectomy, primarily due to an incomplete resection 7. Furthermore, it remains controversial whether external-beam radiotherapy can be used as an adjuvant therapy 8. Recently, a study published in Lancet reported Pexidartinib to be a colonystimulating factor 1 receptor (CSF1R) inhibitor that could be used for the treatment of PVNS; however, the effective rate is only approximately 30% 9. Therefore, there is an urgent need to explore the pathogenesis and identify novel therapeutic targets for PVNS.

Histologically, PVNS consists of fibroblast-like synoviocytes (FLS) and multinucleated giant cells (MGCs), lymphocyte infiltrates, siderophages, and lipid-loaded macrophages 10. Among these, FLS are the predominant cell type comprising the structure of the synovial lining layer and has been proposed to be actively involved in chronic inflammatory reactions during the disease progression of rheumatoid arthritis (RA) and osteoarthritis (OA) 11, 12. Cadherin-11 is a classical molecule that mediates hemophilic cell-to-cell adhesion in FLS and plays an important role in the formation of the normal synovium lining layer 13. FLS have also been reported to be involved in the regulation of inflammation, and cadherin-11-deficient mice are markedly protected against cartilage destruction and exhibit resistance to inflammatory arthritis 14. In RA, studies have shown that cadherin-11 can stimulate FLS to produce inflammatory cytokines and matrix metalloproteinase 15. Furthermore, inhibiting cadherin-11 can reduce the migration and invasion ability of OA FLS, and cadherin-11 antibodies attenuated synovitis and cartilage damage in an animal model of OA 16. However, it remains unclear whether cadherin-11 is involved in the pathogenesis of PVNS and related to the high recurrence, invasion, migration, and cartilage destruction ability of PVNS FLS.

In the present study, we found that inflammatory factors in the synovial fluid of PVNS patients (IL-1β and TNF-α) were significantly up-regulated. Our results also showed cadherin-11 to be highly expressed in the FLSs of PVNS patients, and was positively correlated with recurrence, extra-articular migration, and cartilage destruction. Moreover, inflammatory factors can up-regulate the expression of cadherin-11 to activate the nuclear factor-kappa B (NF-κB) and mitogen-activated protein kinase (MAPKs) signaling pathways, resulting in cartilage destruction. Knocking down cadherin-11 can weaken the migration and invasion ability of PVNS FLS. In addition, inhibition of the PI3K/Akt signaling pathway can down-regulate cadherin-11 in PVNS FLS. These findings demonstrate that cadherin-11 is a key factor in the regulation of PVNS inflammation and is involved in PVNS pathogenesis, which could provide a promising prognostic and therapeutic strategy for PVNS treatment.

## Materials and Methods

### Clinical tissue specimens

A total of 27 synovial tissues from PVNS patients and 13 synovial tissues from anterior cruciate ligament (ACL) injured patients (as a control) were collected under protocols approved by the Ethics Committee of Peking University Third Hospital. Informed consent was obtained from all patients. All synovial tissues were derived from patients who had undergone an arthroscopic synovectomy for further FLS culture and histological testing. The clinical characteristics of these patients are listed in **Table 1.** Macroscopically intact knee cartilage samples from six OA patients were obtained from Peking University Third Hospital at the time of total knee replacement for further chondrocyte culture.

### Histological assessment

The synovial tissues of PVNS and ACL injured patients were excised and fixed in 10% neutral buffered formalin for two days and subsequently dehydrated in a graded ethanol series. After embedding the tissues in paraffin, the samples were cut into 6 μm-thick sections and stained with hematoxylin-eosin (H&E). For immunohistochemistry (IHC), the paraffin sections were incubated with 3% H_2_O_2_ for 15 min to inhibit endogenous peroxidase following by incubation in 10% goat serum for 1 h at room temperature for non-specific antigen blocking. Next, the sections were incubated with antibodies against cadherin-11 (Abcam, Cambridge, MA, USA; 1:200), Ki-67 (Abcam, Cambridge, MA, USA; 1:500), and CD 163 (Abcam, Cambridge, MA, USA; 1:200). The integrated optical density value of positive staining was evaluated by Image J software (National Institutes of Health, USA).

### Knee synovial fluid cytokine chip detection

We selected four different PVNS patients and four different ACL injury patients as our synovial fluid sample source. The synovial fluid was collected from inside the knee joints of four PVNS and ACL injured patients, respectively. The synovial fluid samples were measured using a RayBio® Human Inflammation Antibody Array G-Series 3 AAH-INF-G3 (RayBio, Ray-Biotech, USA). Briefly, the synovial fluid was first dialyzed with dialysis buffer, labeled with biotin, and incubated overnight with arrays. Then the glass slides were incubated with Cy3-conjugated streptavidin for 2 h. Finally, the samples were detected using a Bio-Rad Scanner (Hercules, CA, USA) and the images were analyzed using the RayBio analysis tool.

### Cell isolation and culture

FLS were isolated using an enzymatic dispersion of the synovial tissue from PVNS and ACL injured patients. Briefly, synoviocyte suspensions were prepared from the synovial membranes after mincing and incubating with 1 mg/mL type I collagenase (Invitrogen, CA, USA) in low-glucose Dulbecco's modified Eagle's medium (DMEM) for 2-3 h at 37°C. The samples were then placed in tissue culture dishes in DMEM supplemented with 10% (volume/volume, v/v) fetal bovine serum (FBS, Invitrogen, Australia), 100 U/mL penicillin, and 100 μg/mL streptomycin in a 37 °C, 5% CO_2_ incubator. After the third passage, the non-FLS cells completely disappeared from these culture systems, and the remaining cells were primarily FLS.

For chondrocyte culture, cartilage samples were mechanically sliced into 2-5 mm^3^ pieces and enzymatically digested for 6-10 h in 0.2% type II collagenase (Gibco, USA). The chondrocytes were collected and resuspended in 10% FBS-DMEM/F12 medium (Corning, USA) containing 1 g/L penicillin- streptomycin (Invitrogen, USA) at 37 °C in a humid environment with 5% CO_2_.

### Immunofluorescence analysis

Cultured PVNS FLS were fixed in 4% paraformaldehyde for 15 min and incubated in Triton-X100 (Beyotime Biotechnology) for 10 min to penetrate the cell membrane. Next, a goat serum blocking solution (Beyotime Biotechnology) was added to each well for 1 h. Next, the cells were incubated with rabbit anti-cadherin-11 (Abcam, Cambridge, MA, USA; 1:100) overnight at 4°C and further incubated with fluorescein isothiocyanate (FITC)-conjugated anti-rabbit IgG (Abcam, Cambridge, MA, USA; 1:1000) for 1 h at room temperature in the dark. Finally, the samples were counterstained with phalloidin (Sigma, USA; 1:500) and DAPI (Sigma, USA; 1:1000) for 10 min, and images were captured under a fluorescence microscope (LEICA, USA).

### Cell transfection

siCadherin-11 and scrambled negative control siRNA were obtained from RiboBioCo. Ltd. (Guangzhou, China). The cadherin-11 overexpression plasmid and negative control were obtained from Genechem Co. Ltd. (Shanghai, China). PVNS FLS were transfected with siCadherin-11, a cadherin-11 overexpression plasmid, and negative controls via Lipofectamine 3000 (Invitrogen) according to the manufacturer's instructions. The final plasmid concentration was 100 nM and the final concentration of siRNA was 20 nM.

### Quantitative RT-PCR (qRT-PCR)

The total RNA was obtained from FLS using TRIzol reagent (Invitrogen, Carlsbad, CA, USA). Purified RNA (2 μg) was reverse-transcribed using a RevertAid First Strand cDNA Synthesis Kit (Thermo Fisher Scientific, Boston, MA, USA). Real-time RT-PCR was performed with the Applied Biosystems StepOnePlus Real-Time PCR System (Foster City, CA, USA) with SYBR Green PCR Master Mix (Toyobo, Japan). Glyceraldehyde-3-phosphate dehydrogenase (GAPDH) expression was used to normalize the individual levels of mRNA expression. The primers used in this study are listed in **Table 2.** The relative level of gene expression was expressed as fold-changes as calculated by the 2^-ΔΔCT^ formula. The cells lines used in each test were taken from four different patients, and the experiment used four biological replicates, of which each biological sample tests were technically replicated performed in triplicate.

### Protein extraction and Western blot

PVNS FLS were lysed in radioimmunoprecipitation assay (RIPA) lysis buffer, separated by SDS polyacrylamide gel electrophoresis (PAGE), and transferred to a polyvinylidene fluoride (PVDF) membrane. The membranes were then incubated with corresponding primary antibodies overnight at 4 °C, incubated with secondary antibodies at room temperature for 1 h, and visualized using the BIO-RAD ChemiDoc XRS + system. Anti-phospho-IKKα/β, anti-phospho-IκBα, anti-phospho-p38, anti-phospho-p65, anti-phospho-JNK, and anti-phospho-ERK antibodies were obtained from Cell Signaling Technology (Danvers, MA, USA). Anti-GAPDH, anti-mouse, and anti-rabbit secondary antibodies were purchased from ZSGB-BIO (Beijing, China). The cells lines used in each test were taken from four different patients, and the experiment used four biological replicates, of which each biological sample tests were technically replicated performed in triplicate.

### Wound-healing assay

PVNS FLSs treated according to different experimental conditions were seeded into a 12-well culture plate (5 × 10^5^ cells/well) and cultured to confluence. A horizontal artificial wound was introduced with a P-200 pipette tip in each well. The data for the wounded area were recorded at 0 h and 48 h with a microscope. The cells lines used in each test were taken from three different patients, each patient's sample test was performed in triplicate.

### Cell migration and invasion assay

PVNS FLS were treated according to the different experimental conditions in serum-free DMEM medium and seeded into the upper chamber of the migration or invasion chambers (BD Bioscience, California, USA) in 24-well cell culture plates (2 × 10^4^ cells/well). The lower chamber contained DMEM medium with 10% FBS. After a 48 h incubation, the cells that had not penetrated the filter were removed from the top of the filter using cotton swabs, and the remaining cells were fixed in 4% (w/v) paraformaldehyde for 20 min, stained with 0.1% (w/v) crystal violet, and counted. Migration values were expressed as the number of migrated cells counted under a microscope. Three microscopic fields per membrane in triplicate experiments were counted. The cells lines used in each test were taken from three different patients, each patient's sample test was performed in triplicate.

### Chondrocytes and PVNS FLS co-culture

Human chondrocytes were seeded into a six-well plate and PVNS FLS were seeded into hanging Transwell inserts with complete medium. siCadherin-11, IL-1β, TNF-α, or LY294002 were added to the upper chamber according to different experimental conditions. After co-culturing for 72 h, the total RNA of human chondrocytes was extracted using TRIzol reagent, followed by reverse-transcription and real-time qPCR. The cells lines used in each test were taken from four different patients, and the experiment used four biological replicates, of which each biological sample tests were technically replicated performed in triplicate.

### Statistical analysis

All statistical analyses were performed using SPSS 20.0 (IBM Corp, Chicago, USA). Data were presented as the mean ± standard deviation. Statistical comparisons of two independent groups were achieved using the Shapiro-Wilk test for normality, Levene's test for homogeneity of variance, and a Student's *t*-test. Multiple comparisons were performed using the Shapiro-Wilk test, Levene's test, and one-way analysis of variance (ANOVA) with a post-hoc Bonferroni test. Each n indicates the number of biologically independent samples. Significant data are indicated by *P < 0.05; **P < 0.01; and ***P < 0.001.

## Results

### Inflammatory factors are up-regulated in the synovial fluid of PVNS patients

**Figure 1:**
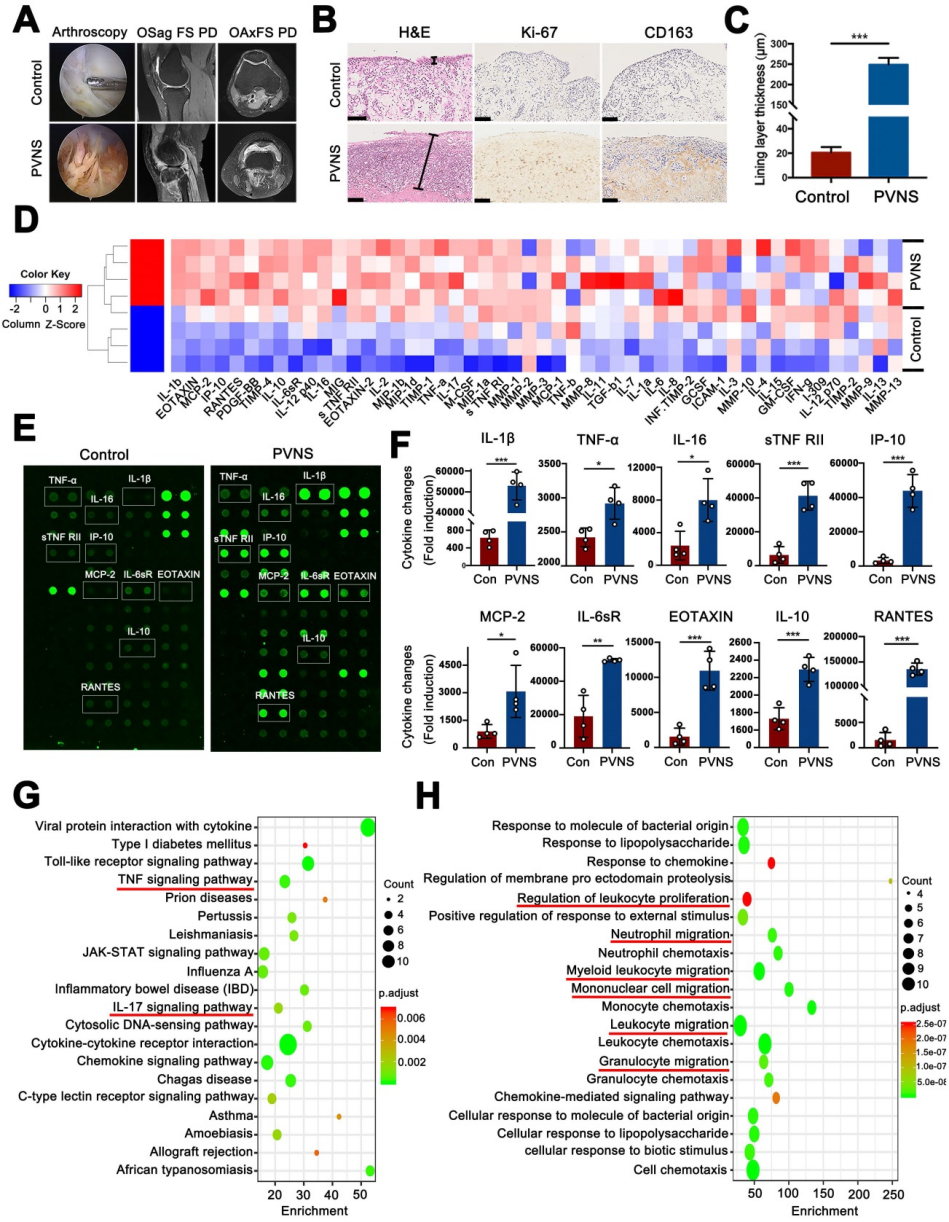
** Inflammatory factors are up-regulated in the synovial fluid of PVNS patients.** (**A**) Arthroscopic observation and MRI features of PVNS. (**B**) Pathological features of PVNS. Scale bar: 100 µm. (**C**) Comparison of the thickness of the synovial lining layer between the PVNS and control group. (**D**) Heat map of the microarray. (**E**) Representative biotin label-based human antibody microarray (RayBio, AAH-INF-G3) images of PVNS and the control group. (**F**) The normalized changes in PVNS cytokine expression in the synovial fluid of each group based on the microarray results. (**G-H**) Pathway (G) and GO (H) analysis of the microarray. *P < 0.05; **P < 0.01; ***P < 0.001.

In this study, we first observed the general characteristics of PVNS via arthroscopy and MRI. Arthroscopic images showed that the synovial tissue of PVNS patients was reddish brown due to hemosiderosis and were significantly hyperplastic compared with the control group. MRI revealed that the synovial tissue of PVNS displayed a high signal in the FS-PD sequence. The knee joint of PVNS patients presented with increased severe joint swelling and cartilage degeneration (**Figure 1A**). To identify the pathological characteristics of PVNS, histological and immunohistochemical staining were performed. H&E staining showed that the PVNS synovial lining layer was obviously thickened (**Figure 1B**-**C**). The immunohistochemical analysis revealed that positive Ki-67 and CD163 staining was significantly increased (**Figure 1B**).

To further investigate changes in the articular microenvironment caused by PVNS, the levels of cytokine expression in the synovial fluid of both the control group (ACL injured patients, n = 4) and PVNS group (knee PVNS patients, n = 4) were evaluated using a human antibody array. The array results showed that compared with the control group, the expression of 45 inflammatory factors were upregulated in the PVNS group (**Figure 1D-H**) (IL-1β, TNF-α, IL-16, and IL-10). Moreover, the results of the pathway analysis showed that the TNF-α signaling pathway, IL-17 signaling pathway, and other inflammatory pathways were activated (**Figure 1G**). A GO analysis showed that these inflammatory factors were primarily involved in regulating the leukocyte proliferation and migration capacity of leukocyte, neutrophil, mononuclear cell and granulocyte (**Figure 1H**). Thus, PVNS disease can induce a highly inflammatory microenvironment in the joints, and the PVNS diseased tissue itself can also be affected by this inflammatory environment.

### Cadherin-11 is highly expressed in PVNS and promoted the invasion and migration of PVNS FLS

**Figure 2:**
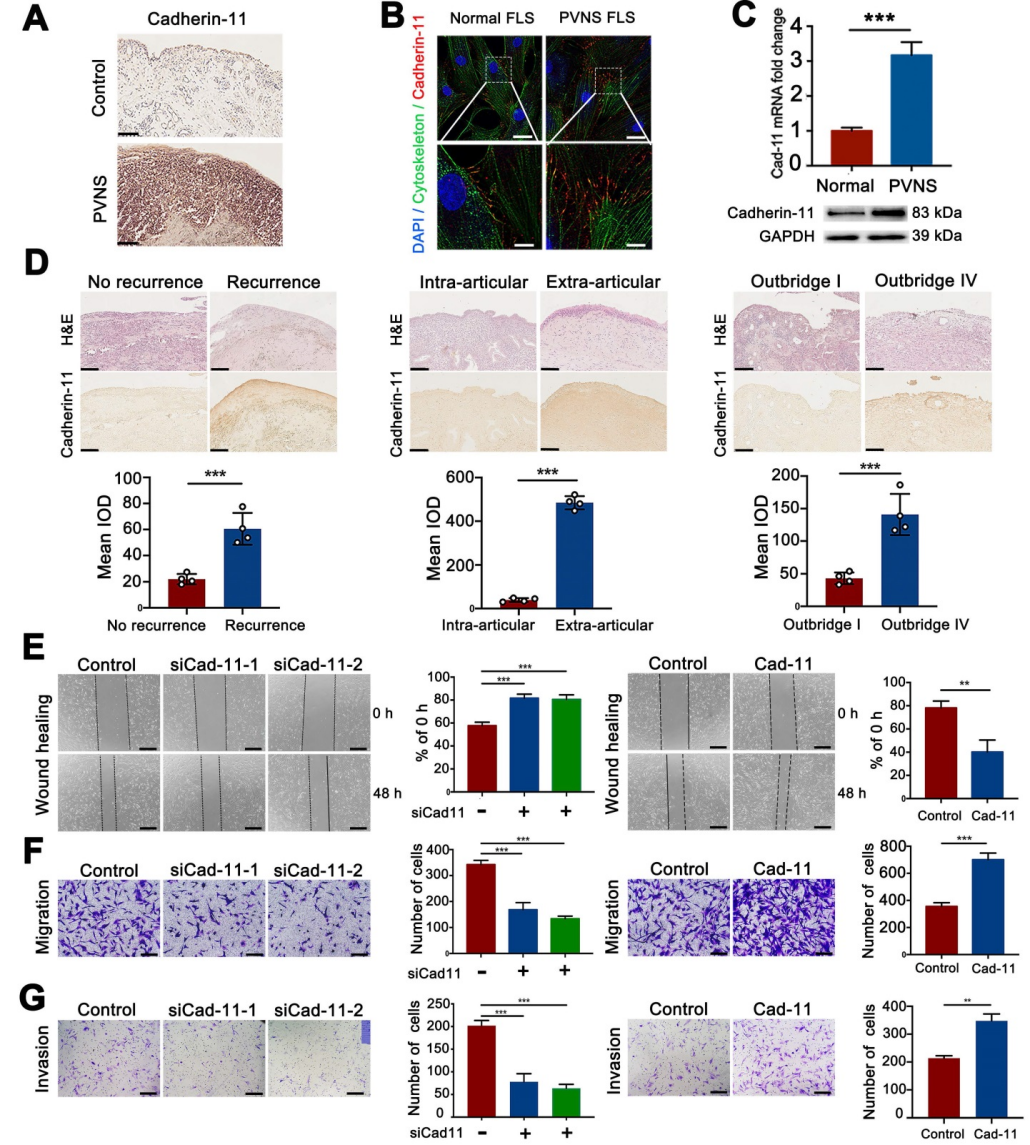
** Cadherin-11 is highly expressed in PVNS and promotes the invasion and migration of PVNS FLS.** (**A**) Cadherin-11 immunohistochemistry image of PVNS and the control group. Scale bar: 100 µm. (**B**) Cadherin-11 immunofluorescence image of PVNS FLS. Red: cadherin-11, Green: cytoskeleton, Blue: nucleus. Scale bar: 20 µm and 5 µm. (**C**) Cadherin-11 qRT-PCR and Western blot analysis of PVNS FLS and the control group. (**D**) H&E and cadherin-11 immunohistochemistry image of each group of PVNS patients. Scale bar: 100 µm. (**E**) Wound-healing analysis of PVNS FLS. Scale bar: 50 µm. (**F-G**) Transwell migration (F) and invasion (G) assays of PVNS FLS. Scale bar: 50 µm. *P < 0.05; **P < 0.01; ***P < 0.001.

To detect the expression of cadherin-11 in PVNS, tissue immunohistochemical staining, cell immunofluorescence analysis**,** qRT-PCR, and a Western blot were performed. Immunohistochemical staining revealed that the cadherin-11 was strongly positive and significantly increased compared with the control group (**Figure 2A**). The qRT-PCR, Western blot, and immunofluorescence results showed that cadherin-11 in PVNS FLS was increased at both the mRNA and protein level. Moreover, cadherin-11 was mainly expressed at the junction between cells, which highly coincides with the cytoskeleton (**Figure 2B**-**C**). Moreover, we also found that cadherin-11 expression differed between PVNS patients with or without recurrence and extra-articular invasion, as well as in patients with mild (Outbridge I) or heavier (Outbridge IV) cartilage damage. Cadherin-11 expression in PVNS patients who experienced recurrence, extra-articular invasion, and heavier cartilage damage was higher than that of the corresponding control group. These findings revealed that cadherin-11 was correlated with PVNS disease behavior and could be used as a molecular marker to predict PVNS progression (**Figure 2D**).

To further explore the molecular biological function of cadherin-11, we constructed a cadherin-11 overexpression plasmid and specific knockdown siRNA. qRT-PCR and Western blot were performed for verification that the plasmid and siCadherin-11 could regulate the expression of cadherin-11 at both the mRNA and protein level (**Figure S1**). Wound-healing and Transwell assays were performed to further study whether the migration and invasion ability of PVNS FLS were affected by cadherin-11. These results indicate that the migratory and invasive capacity of siCadherin-11 transfected PVNS FLS was markedly reduced. In addition, after transfection with a cadherin-11 overexpression plasmid, the migratory and invasive capacity of PVNS FLS were significantly increased (**Figure 2E**-**G**). Together, cadherin-11 can regulate the migration and invasion of PVNS FLS.

### Inflammatory environment up-regulated cadherin-11 and further promoted the migration and invasion of PVNS FLS

**Figure 3:**
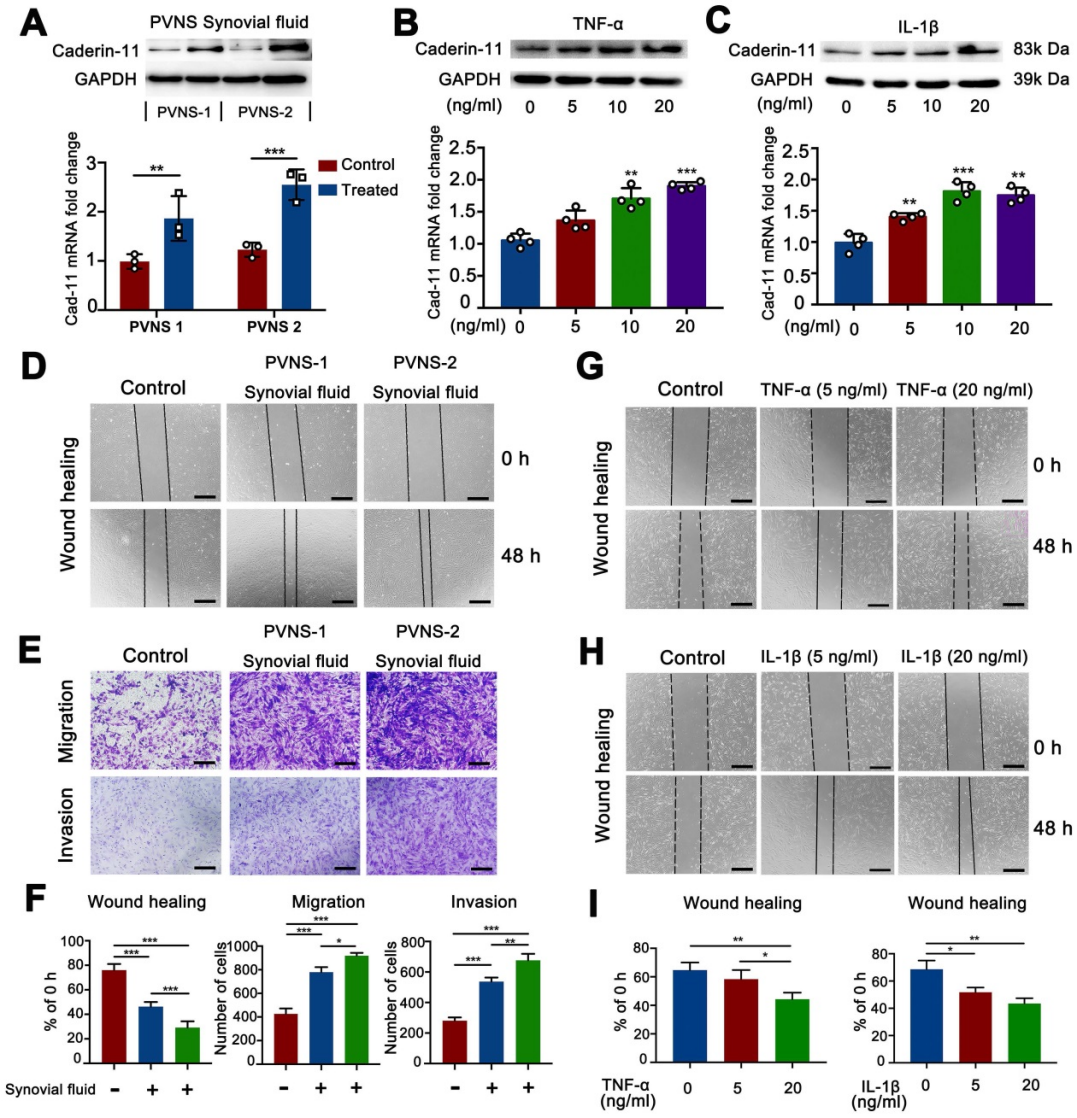
** Inflammatory environment up-regulated cadherin-11 and further promoted the migration and invasion of PVNS FLS.** (**A**) Cadherin-11 qRT-PCR and Western blot analysis of normal FLS under PVNS synovial fluid stimulation. (**B-C**) Cadherin-11 qRT-PCR and Western blot analysis of PVNS FLS under TNF-α (A) or IL-1β (B) stimulation with different doses (5, 10, or 20 ng/mL). (**D**) Wound-healing analysis of normal FLS under PVNS synovial fluid stimulation. (**E**) Transwell migration and invasion assays of normal FLS under PVNS synovial fluid stimulation. (**F**) Quantitative analysis results of Wound-healing and Transwell assays of normal FLS under PVNS synovial fluid stimulation. (**G-H**) Wound-healing analysis of PVNS FLS under TNF-α (G) or IL-1β (H) stimulation with different doses (0, 5, or 20 ng/mL). (**I**) Quantitative analysis results of Wound-healing assays of PVNS FLS under TNF-α or IL-1β stimulation. Scale bar: 50 µm.

The above experimental results showed that PVNS was associated with a highly inflammatory environment. To further explore the effect of inflammatory environment on cadherin-11 expression in PVNS FSL, we first collected the synovial fluid of two PVNS patients for cell stimulation experiments, Western blot and real-time qPCR analyses showed that the synovial fluid of PVNS patients could increase cadherin-11 expression at both the mRNA and protein level (**Figure 3A**). Then, we selected IL-1β and TNF-α, which were representative and obvious factors in the inflammatory factor array for further stimulation experiments. Western blot and real-time qPCR analyses showed that both IL-1β and TNF-α (5, 10, or 20 ng/mL) could increase cadherin-11 expression at both the mRNA and protein level. There was also a trend of increased cadherin-11 expression with the dosage of IL-1β and TNF-α, although there were no statistical differences among the varying concentrations (**Figure 3B**-**C**).

Since inflammatory environment can up-regulate cadherin-11 expression, we further verified whether PVNS synovial fluid, IL-1β and TNF-α could promote the migration and invasion ability of PVNS FLS. Wound-healing assays showed that PVNS synovial fluid, IL-1β (5 and 20 ng/mL) and TNF-α (20 ng/mL) could promote the migratory ability of PVNS FLS (**Figure 3D and G**-**I**). Transwell results also showed that PVNS synovial fluid could promote the invasion and migration ability of PVNS FLS (**Figure 3E**-**F**). These results suggest that inflammatory environment, especially the IL-1β and TNF-α, may promote PVNS FLS migration by up-regulating cadherin-11, which was dependent on a certain concentration.

### Cadhein-11 cooperates with inflammatory factors to activate the NF-κB and MAPK signaling pathways in PVNS FLS

**Figure 4:**
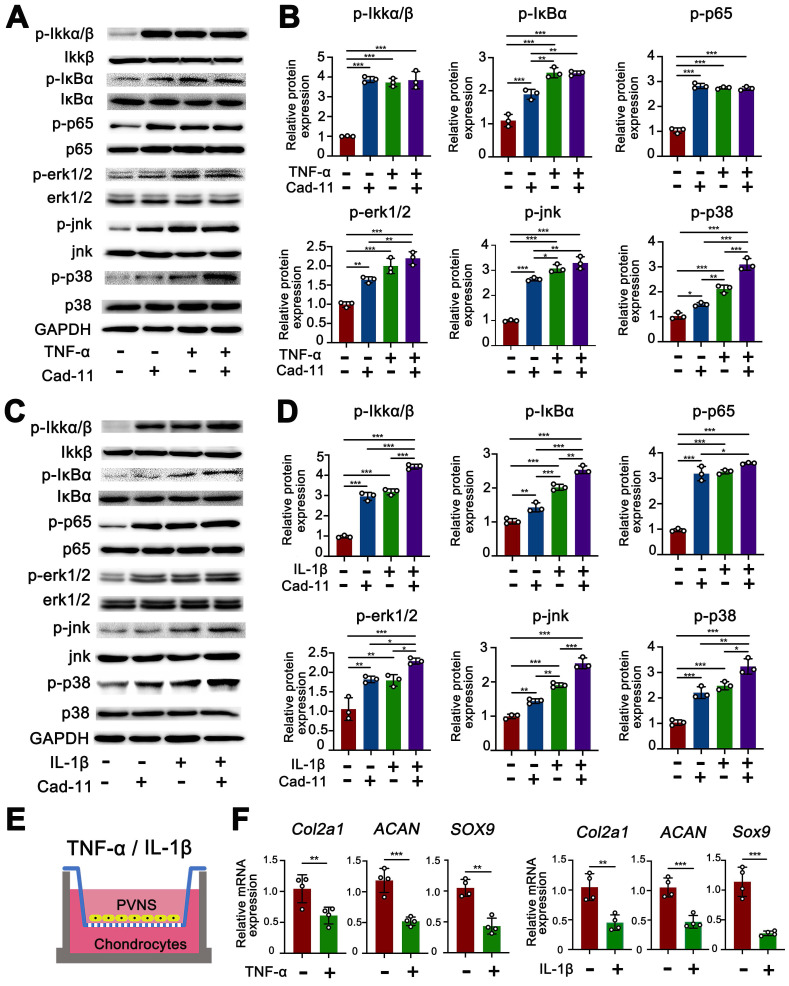
** Cadhein-11 cooperates with inflammatory factors to activate the NF-κB and MAPK signaling pathways in PVNS FLS.** (**A**) Western blot analysis of NF-κB and MAPK signaling pathways activated by Cadherin-11 cooperated with TNF-α in PVNS FLS. (**B**) The quantification Western blot analysis of NF-κB and MAPK signaling pathways activated by Cadherin-11 cooperated with TNF-α. (**C**) Western blot analysis of NF-κB and MAPK signaling pathways activated by Cadherin-11 cooperated with IL-1β in PVNS FLS. (**D**) The quantification Western blot analysis of NF-κB and MAPK signaling pathways activated by Cadherin-11 cooperated with IL-1β. (**E**) Schematic diagram of the co-culture system consisting of chondrocytes and PVNS FLS with or without TNF-α and IL-1β. (**F**) The level of mRNA expression of classic ECM-related genes in chondrocytes within the co-culture system after 72 h.

The MAPK family and NF-κB are well-characterized inflammatory signaling pathways that participate in the process of synovitis 17. Thus, there is a need to further investigate whether these signaling pathways are involved in the disease process of PVNS and explore the function of cadherin-11. We first tested if the MAPK and NF-κB pathways are activated by cadherin-11 in PVNS FLS and explored whether cadherin-11 plays a synergistic role in inflammatory factors. Members of the MAPK and NF-κB pathways were detected by a Western blot. The results showed that NF-κB family members (IKK, IκBα, and p65) phosphorylation, as well as MAPK family members (JNK, ERK1/2, and p38) phosphorylation in PVNS FLS were significantly activated by stimulation with inflammatory factors (IL-1β and TNF-α each 20 ng/mL) and cadherin-11 overexpression plasmid transfection, respectively. In addition, simultaneous inflammatory factor stimulation and overexpression of cadherin-11 could further activate IKKα/β, IκBα, ERK1/2, JNK, and p38 phosphorylation, indicating that cadherin-11 played a synergistic role with IL-1β and TNF-α in activation of inflammatory pathways (**Figure 4A-D**). Quantification analysis and statistics of Western blot results were presented in **Figure 4B and D**.

Next, we tested whether inflammatory factors can activate PVNS FLS through the MAPK and NF-κB signaling pathways to cause chondrocyte degeneration. The results of the co-culture assay revealed that the expression of COL2A1, SOX9, and ACAN in chondrocytes were down-regulated by PVNS FLS following IL-1β and TNF-α (20 ng/mL) stimulation (**Figure 4E-F**). In addition to high-dose concentrations of inflammatory factors that can activate PVNS FLS to cause chondrocytes degeneration, the results also showed that low-dose IL-1β (5 ng/mL) can also cause down-regulation of the expression of chondrocyte extracellular matrix related genes (**Figure S2A**-**B**).

### Inhibition of cadherin-11 prevented the inflammatory factor-mediated invasion, migration and chondrocyte degeneration in PVNS FLS

**Figure 5:**
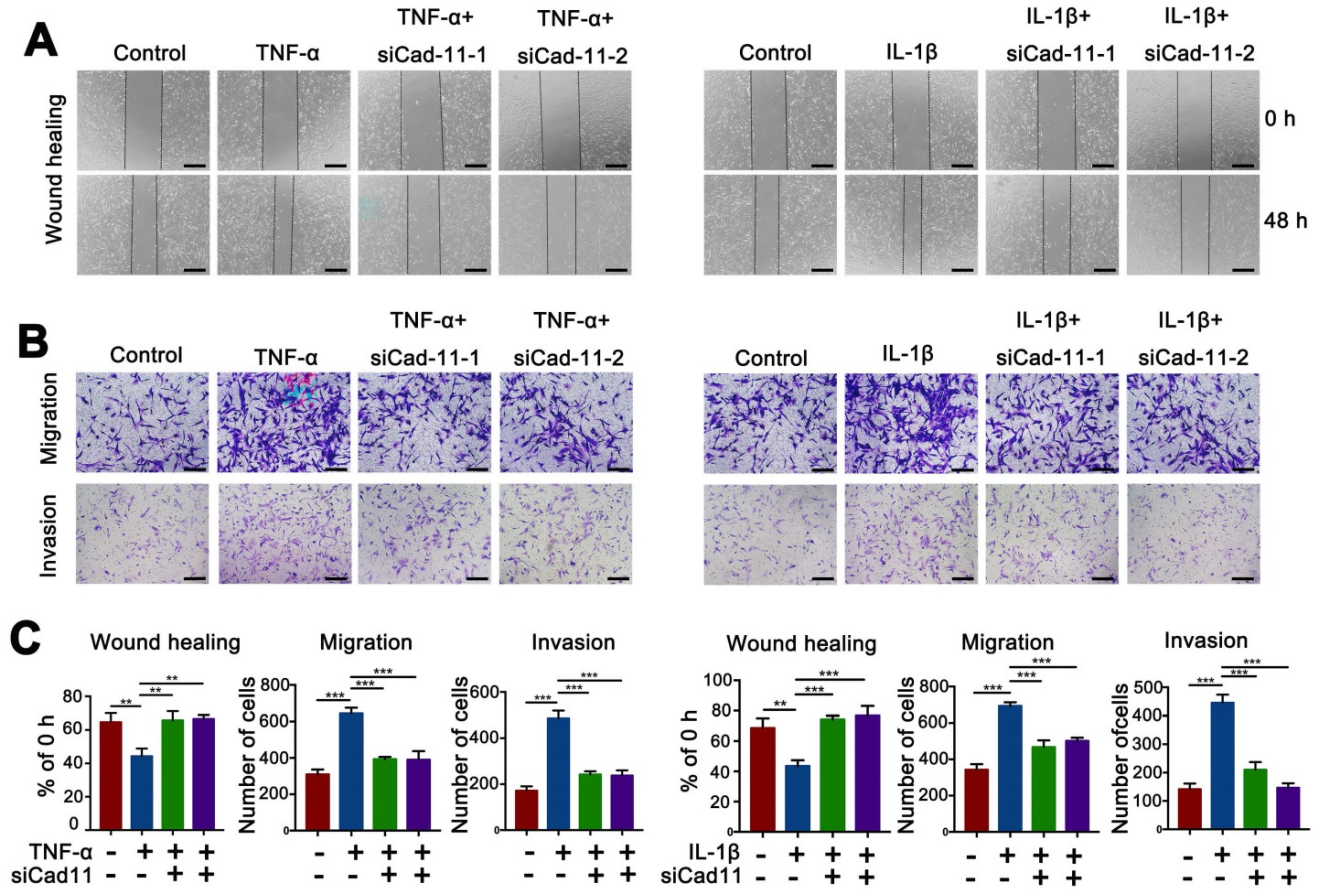
** Inhibition of cadherin-11 prevented the inflammatory factor-mediated invasion and migration of PVNS FLS.** (**A**) Wound-healing assays of PVNS FLS under inflammatory factors (TNF-α or IL-1β) stimulation or simultaneously transfected with siCadherin-11. Scale bar: 50 µm. (**B**) Transwell assays of PVNS FLS under inflammatory factors (TNF-α or IL-1β) stimulation or simultaneously transfected with siCadherin-11. Scale bar: 50 µm. (**C**) The statistical results of the wound-healing and Transwell assays.

**Figure 6:**
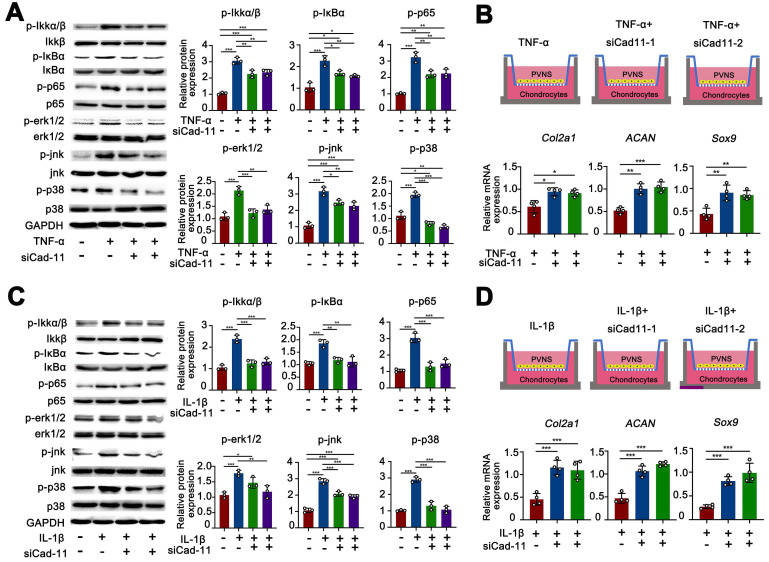
** Inhibition of cadherin-11 prevented the inflammatory factor-activated phosphorylation of NF-κB and MAPK signaling pathways in PVNS FLS.** (**A**)The western blot analysis of TNF-α activated phosphorylation of NF-κB and MAPK signaling pathways prevented by siCadherin-11. (**B**) Schematic diagram of the co-culture system consisting of chondrocytes and PVNS FLS with TNF-α or siCadherin-11. The level of mRNA expression of classic ECM-related genes in chondrocytes within the co-culture system after 72 h. (**C**) The quantification Western blot analysis of IL-1β activated phosphorylation of NF-κB and MAPK signaling pathways prevented by siCadherin-11. (**D**) Schematic diagram of the co-culture system consisting of chondrocytes and PVNS FLS with IL-1β or siCadherin-11. The level of mRNA expression of classic ECM-related genes in chondrocytes within the co-culture system after 72 h. *P < 0.05; **P < 0.01; ***P < 0.001.

Our results confirmed that inflammatory factors could up-regulate cadherin-11 expression and further promote the migration and migration of PVNS FLS. Inflammatory factors could also activate PVNS FLS through the MAPK and NF-κB signaling pathways to cause chondrocytes degeneration. To further investigate the biological function of cadherin-11 in the above process, we knocked down cadherin-11 via siRNA transfection while stimulating with inflammatory factors. The wound-healing and Transwell results showed that cadherin-11 inhibition prevented the IL-1β (20 ng/mL) and TNF-α (20 ng/mL)-mediated invasion and migration of PVNS FLS (**Figure 5A**-**C**). The Western blot results showed that siCadherin-11 could partially block IL-1β and TNF-α induced IKK, IκBα, and p65 phosphorylation, as well as JNK, ERK1/2, and p38 phosphorylation in PVNS FLS (**Figure 6A and C**). The co-culture assays results revealed that COL2A1, SOX9, and ACAN expression was up-regulated following the inhibition of cadherin-11 (**Figure 6B and D**). Those finding indicated that the inhibition of cadherin-11 expression could block the activation of the MAPK and NF-κB signaling pathways activated by IL- 1β and TNF-α in PVNS FLS to prevent chondrocyte degeneration. In summary, cadherin-11 inhibition prevented the inflammatory factor-mediated invasion, migration, and chondrocyte degeneration of PVNS FLS.

### Inflammatory factors up-regulated cadherin-11 through activation of the PI3K/Akt pathway

**Figure 7:**
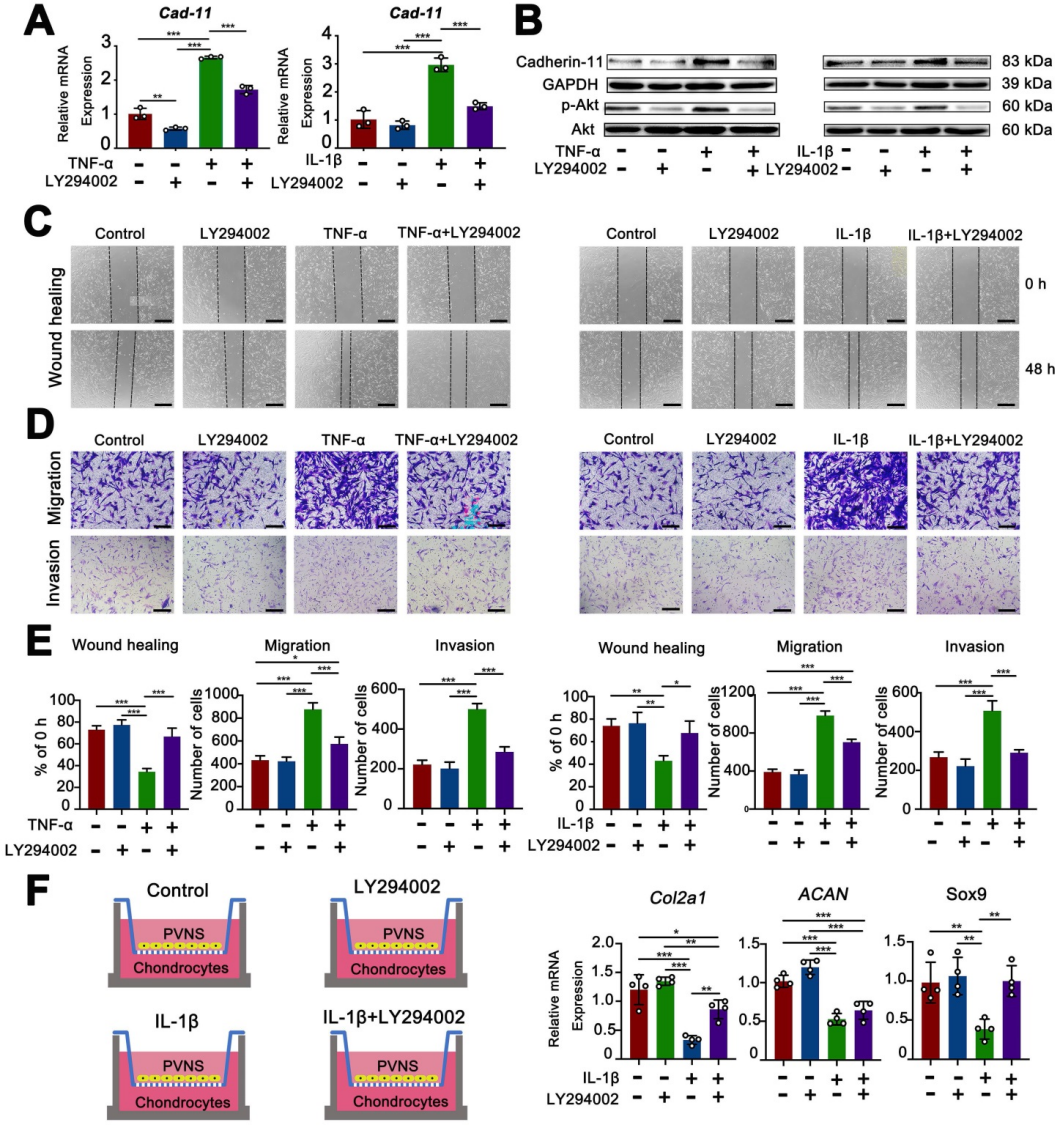
** Inflammatory factors upregulate cadherin-11 through activation of the PI3K/Akt pathway, whereas inhibition of PI3K/Akt pathway decreased the migration, invasive, and cartilage destruction capacity of PVNS FLS induced by inflammatory factors.** (**A-B**) qRT-PCR and Western blot analysis of the expression of cadherin-11 and P-Akt in PVNS FLS induced by inflammatory factors (TNF-α or IL-1β) with or without the PI3K inhibitor, LY294002. (**C-E**) Wound-healing and Transwell assays were performed in PVNS FLS induced by inflammatory factors (TNF-α or IL-1β) with or without the PI3K inhibitor, LY294002. Statistical results of wound-healing and Transwell assays are shown accordingly. (**F**) The schematic diagram of the co-culture system consisted of chondrocytes and PVNS FLS with or without IL-1β and LY294002. The level of mRNA ex=pression of classic ECM-related genes in chondrocytes within the co-culture system after 72 h. *P < 0.05; **P < 0.01; ***P < 0.001.

The PI3K/Akt pathway is well known to be a classic signaling pathway involved in the regulation of various functions, including cell survival, proliferation, invasion, and migration 18. The experimental results described above showed that cadherin-11 was up-regulated by inflammatory factors. To further explore the associated mechanism, we used both a Western blot and real-time qPCR analysis to detect changes in the PI3K/Akt pathway during these processes. The results showed that cadherin-11 was increased at both the mRNA and protein level in PVNS FLS following IL- 1β (20 ng/mL) and TNF-α (20 ng/mL) stimulation compared to the control group. IL-1β and TNF-α stimulation could also increase the phosphorylation-dependent activation of PI3K/Akt. When we used the specific PI3K inhibitor, LY294002, to pre-treat PVNS FLS before inflammatory factor stimulation, the expression of cadherin-11 and phosphorylation of Akt were significantly attenuated. These findings indicate that the up-regulation of cadherin-11 by inflammatory factors may be mediated through the PI3K/Akt pathway (**Figure 7A**-**B**).

### Inhibition of the PI3K/Akt pathway decreased the migratory, invasive, and cartilage destruction capacity of PVNS FLS induced by inflammatory factor

To investigate the effect of PI3K/Akt inhibitor (LY294002) on the invasion, migration, and cartilage destruction ability of PVNS FLS in wound-healing, Transwell, and co-culture assays were performed. The wound-healing and Transwell results showed that LY294002 inhibited the IL-1β (20 ng/mL) and TNF-α (20 ng/mL)-mediated invasion and migration of PVNS FLS (**Figure 7C**-**E**). The results of the co-culture assay displayed that the expression of COL2A1, SOX9, and ACAN could return to the same level of expression as before IL- 1β stimulation (**Figure 7F**).

In addition, we further explored whether the PI3K/Akt inhibitor (LY294002) can inhibit the increased invasion and migration of PVNS FLS caused by cadherin-11 overexpression, the results showed that LY294002 could not decreased the migration and invasive capacity of PVNS FLS induced by cadherin-11 overexpression plasmid (**Figure S3A**-**B**). This may be because cadherin-11 directly regulates the cell-to-cell connection, thereby promoting the invasion and migration of PVNS FLS. PI3K/Akt inhibitor only cuts off the positive feedback association between inflammation and cadherin-11, but cannot directly inhibit the effect of cadherin-11 itself.

## Discussion

PVNS is a rare tumor-like hyperplastic disease that occurs in the synovial tissue. Based on the scope of its pathological damage, the following two forms are described: 1) diffuse PVNS (DPVNS); or 2) localized PVNS (LPVNS) 19. DPVNS most frequently affects the large joints and the knee is involved in 66% - 80% of cases, with a recurrence rate higher than LPVNS 20. Persistent disease can cause cartilage destruction and bone erosion, resulting in joint functional limitations and long-term pain. Additionally, DPVNS may cause extra-articular invasion, eventually leading to joint replacement or even amputation 21. Therefore, this study used DPVNS as the main research object. General observations of PVNS under arthroscopy revealed a hypertrophic synovial process characterized by villous, nodular, and villonodular proliferation and pigmentation (**Figure 1A**). Our histological results showed that the PVNS synovial lining layer was significantly thickened, which was approximately 100 times thicker than that of the control group. Although the current diagnosis of PVNS mainly depends on pathology, its specific molecular markers are still controversial, which primarily includes Ki-67, CD163, CD55, CSF-1, and CSF-1R 22, 23. Our immunohistochemical results showed a positive expression of CD163 and Ki-67, indicating increased mononuclear macrophage aggregation and enhanced PVNS FLS proliferation ability respectively, which was consistent with previous reports 24.

The pathogenesis of PVNS is unclear and has been associated with controversy. While some scholars consider PVNS to be a chronic inflammatory disease 5, others believe it to be an abnormally proliferative tumorous lesion caused by genetic mutation 6. Due to incomplete research on the pathogenesis of PVNS, the associated treatment has been primarily limited to surgery synovectomy under arthroscopy 25. However, for DPVNS, it is difficult to completely remove the diseased synovium, which results in a poor prognosis and easy recurrence, at a rate of approximately 21% - 50% 7. Due to the high rate of recurrence of surgery, postoperative radiotherapy may often be used as an adjunct therapy, but has certain side effects and its usage is controversial 7. Recently, targeting CSF-1R with either small molecules or antibodies has yielded interesting results *in vitro* for PVNS treatment but exhibits limited antitumor activity *in vivo* 26. Therefore, further understanding of PVNS pathogenesis and an exploration of novel therapeutic targets for patients is urgently required.

Due to the controversy surrounding whether PVNS is an inflammatory or a neoplastic disease, we used an antibody array to explore the microenvironment of the PVNS joint fluid. The results showed that various inflammatory factors were up-regulated, especially IL-1β, which was approximately 60 times higher than that of the control group. Although PVNS was originally termed a synovitis, this is the first study to detect the expression of inflammatory factors in the joint fluid. IL-1β and TNF-α are classic inflammatory factors which have been reported to participate in OA and RA synovium inflammation and are involved in driving the inflammatory cascade both independently or in conjunction with other cytokines 27. Our results show that these cytokines are also involved in the pathogenesis of PVNS and participate in the regulation of PVNS invasion and migratory functions. This indicates that PVNS disease can cause the joints to be in a highly inflammatory microenvironment, which can also affect the PVNS synovial tissue itself. This provides a theoretical basis for treating PVNS by targeting inflammation.

Cadherin-11 as a classical cell adhesion molecule responsible for tissue morphogenesis and synovial architecture mediating contact between FLS and organization in the lining layer 13. It has recently been reported that cadherin-11 has an essential function in synovial inflammation and arthritis pathology 15. David reported that cadherin-11-deficient mice display a disorganized synovial reaction to inflammation and are resistant to inflammatory arthritis 14. In RA, research shows that cadherin-11 plays a central role for rheumatoid pannus formation and can be a therapeutic target for the amelioration of autoimmune experimental arthritis 28. However, the expression of cadherin-11 in PVNS synovial tissue and whether it is involved in the pathogenesis of PVNS is unclear. Our data reveal that cadherin-11 immunohistochemical staining of the PVNS synovial tissue was strongly positive compared to that of the control group and cadherin-11 expression was increased in PVNS FLS. The main clinical feature of PVNS, which is also the greatest difference compared to other synovitis (OA or RA), is its high recurrence rate, invasion, migration, and cartilage destruction ability, as well as the possibility of malignant transformation 29. However, it is unclear whether cadherin-11 is related to those features of PVNS. In the present study, cadherin-11 expression in PVNS patients who exhibited recurrence, extra-articular invasion, and heavier cartilage damage was higher than that of the corresponding control group. We also found that cadherin-11 participates in regulating the migration and invasion ability of PVNS FLS, cadherin-11 overexpression can increase the migration and invasion ability of PVNS FLS and knock down can play the opposite function, which may be related to fibronectin adhesion mediated by cadherin-11/syndecan-4 complexes 30. The above experimental results reveal that cadherin-11 is correlated with PVNS disease behavior and can be used as a molecular marker to predict disease progression.

The above experimental results confirm that both cadherin-11 and inflammatory factors play a critical role in PVNS pathogenesis. Thus, we hypothesize that high levels of cadherin-11 expression mediated by inflammatory factors in PVNS can also regulate the activation of inflammatory pathways and participate in PVNS pathogenesis. To further study the associated mechanism, we established a crosslink interaction between inflammatory factors and cadherin-11. Thus, using qPCR and a Western blot, we confirmed that both IL-1β and TNF-α could increase cadherin-11 expression. There was also a trend of increased cadherin-11 expression with the dosage of IL-1β and TNF-α. We also found that IL-1β and TNF-α can activate PVNS FLS and significantly increase the migration and invasion ability of PVNS FLS, which is consistent with previous reports of inflammatory factors in OA, RA, and tumor diseases 31, 32. At the same time, cadherin-11 could significantly activate the phosphorylation of the NF-κB and MAPK signaling pathways in PVNS FLS. Moreover, there were dramatic synergistic effects between cadherin-11 with TNF-α or IL-1β for activation of the NF-κB and MAPK pathways, suggesting that cadherin-11 might set a threshold for PVNS FLS responding to activation by inflammatory cytokines. Sook Kyung Chang et. al. previously reported that cadherin-11 directly modulates IL-6 production and engagement-induced FLS to produce other inflammatory factors 15. This forms a positive feedback loop of inflammatory factors-cadherin-11-inflammatory factors in PVNS FLS, leading to an accumulation of inflammatory factors in the joint fluid, uninterrupted up-regulation of cadherin-11 in PVNS FLS, and the continuous activation of inflammatory pathways. Such effects further lead to an increased migration and invasion ability of PVNS FLS, thereby promoting the recurrence, extra-articular metastasis of PVNS, and erosive destruction of the cartilage. In addition, we found that the inhibition of cadherin-11 expression could block the activation of the above pathways, decrease the migration and invasion ability of PVNS FLS, and reduce chondrocyte damage which were activated by IL-1β and TNF-α. This illustrates that the down-regulate cadherin-11 could cut off this positive feedback loop to interfere with PVNS disease development.

Although the results confirmed that IL-1β and TNF-α could up-regulate cadherin-11 expression, the specific molecular mechanism remains unclear. The PI3K/Akt pathway has been well-established as a classic signaling pathway involved in the regulation of various functions (cell survival, proliferation, invasion, and migration) 18. A study conducted by Alkhadar reported that nerve growth factors were triggered by the PI3K/Akt pathway in oral and salivary tumor cells and induced oral and salivary tumor cells scattering and migration 33. Moreover, activation of PI3K/Akt pathway is closely related to inflammatory stimulation. Feldman has demonstrated that the PI3K/Akt pathway can be activated by several cytokines (TNF-α in RA synoviocytes) 34. Our previous research also confirmed that inflammatory factors can activate the PI3K/Akt pathway in OA FLS 16. In the current study, the results showed that IL-1β or TNF-α up-regulated the expression of cadherin-11 in PVNS FLS through the PI3K/Akt pathway. Moreover, inhibition of the PI3K/Akt pathway was shown to down-regulate the inflammatory factors mediated by the up-regulation of cadherin-11. Given the important role of the PI3K/Akt pathway in promoting inflammation, cell proliferation, invasion, and migration, several studies have investigated the effects of a pharmacological inhibition of the PI3K/Akt pathway in inflammatory joint disease. TASP0415914, a known PI3Kγ inhibitor has been reported to reduce the symptoms of collagen-induced arthritis 35. Haruta also reported that the use of ZSTK474 as a pan-class I PI3K inhibitor could improve inflammation and disease progression in RA 36; however, the application of PI3K/AKT inhibitors on PVNS treatment have not been previously reported. As a specific inhibitor of the PI3K/Akt pathway, LY294002 can simultaneously inhibit PI3Kα/δ/β and has been reported in RA treatment 37. Our results show that LY294002 can inhibit both the IL-1β and TNF-α-mediated migration and invasion of PVNS FLS and chondrocyte damage caused by activated PVNS FLS in a co-culture system. Therefore, there is possibility that PI3K/Akt inhibitors can be used for the treatment of PVNS.

**Figure 8:**
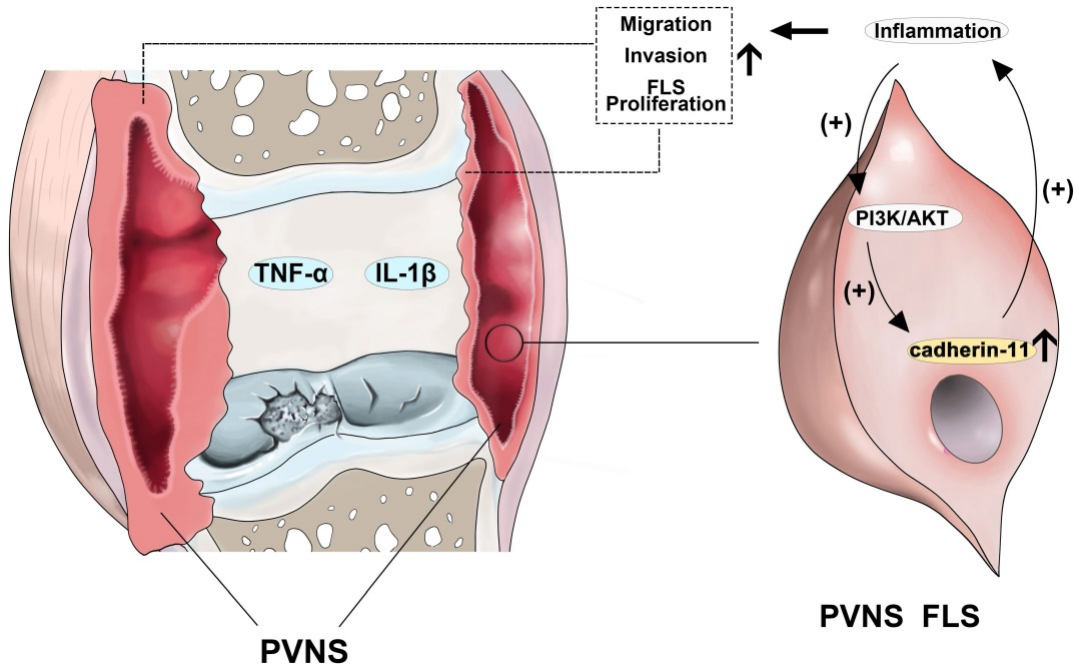
Schematic illustration of cadherin-11 cooperates with inflammatory factors in the pathogenesis of PVNS.

Previous studies on the mechanism of PVNS-associated inflammation are limited to the high expression of inflammatory factors and matrix metalloproteinases in the synovial tissue itself, and have been shown to participate in the process of cartilage destruction 38. However, this does not explain the tumor characteristics of PVNS, such as recurrence, metastasis, and malignant transformation, which our present research addresses. In brief, inflammatory factors can up-regulate cadherin-11 through the PI3K/Akt pathway, forming a positive feedback loop and activating the NF-κB and MAPK pathways. This process continuously promotes the migration and invasion of PVNS FLS, eventually causing relapse, metastasis, and cartilage damage (**Figure 8**). Indeed, inflammatory mediators are important components of the local tumor microenvironment. In some types of cancer, inflammation exists prior to malignant changes, and carcinogenic events can also induce an inflammatory microenvironment that promotes tumor development 39. Under the cooperative regulation of inflammatory factors and cadherin-11, the invasiveness and proliferation of PVNS FLS gradually loses control, eventually promoting the transformation of PVNS from an initial inflammatory disease to neoplastic disease. Treatment by targeting the inflammation or cadherin-11 may be an exciting new era for PVNS therapies.

There are some limitations associated with our research, primarily because there is no suitable animal model for PVNS, and the experiment was mainly carried out at the cellular level *in vitro*. The treatment of PVNS by targeting the inflammation or cadherin-11 still remains to be verified *in vivo*.

## Conclusion

In summary, our findings demonstrate that PVNS is an inflammatory disease, and cadherin-11 plays an important role in the pathogenesis of PVNS. Cadherin-11 forms a positive feedback loop with inflammatory factors, which further activated the NF-κB and MAPK pathways and triggered the inflammatory waterfall effect. This resulted in PVNS with a high recurrence, high invasiveness, and strong cartilage destruction ability. Under the cooperative regulation of inflammatory factors and cadherin-11, the invasiveness and proliferation of PVNS FLS gradually loses control, eventually promoting the transformation of PVNS from an initial inflammatory disease to neoplastic disease. Thus, inhibiting the inflammatory factors-induced by cadherin-11 may represent a new therapeutic strategy for PVNS treatment.

## Supplementary Material

Supplementary figures and tables.

## Figures and Tables

**Table 1:** Clinical characteristics of PVNS and ACL injured patients

Characteristics	PVNS	Control (ACL Injured)
Patients, n	27	13
**Gender**		
Male	10	11
Female	17	2
**Age**	38.6±11.3	30.2±9.7
**Location**		
Knee	22	13
Elbow	4	0
Ankle	1	0
**Recurrence**		
Yes	7	NA
No	20	NA
**Extra-articular**		
Yes	6	NA
No	21	NA

**Table 2:** Primer sequences for real-time PCR

Gene name		Sequences
Cadherin-11	F	5′-GATCGTCACACTGACCTCGACA-3′
	R	5′-CTTTGGCTTCCTGATGCCGATTG-3′
Col2a1	F	5′-CCAGATGACCTTCCTACGCC-3′
	R	5′-TTCAGGGCAGTGTACGTGAAC -3′
Acan	F	5′-ACTCTGGGTTTTCGTGACTCT-3′
	R	5′-ACACTCAGCGAGTTGTCA TGG -3′
Sox9	F	5′-AGCGAACGCACA TCAAGAC -3′
	R	5′-CTGTAGGCGATCTGTTGGGG -3′
GAPDH	F	5′-GCACCGTCAAGGCTGAGAAC -3′
	R	5′-TGGTGAAGACGCCAGTGGA -3′

## Abbreviations

ACL: anterior cruciate ligament
CSF1R: colonystimulating factor 1 receptor
FLS: fibroblast-like synoviocytes
MAPK: mitogen-activated protein kinase
NF-κB: nuclear factor-kappaB
OA: osteoarthritis
PVNS: pigmented villonodular synovitis
qRT-PCR: quantitative real-time polymerase chain reaction
RA: rheumatoid arthritis
TGCT: diffuse-type tenosynovial giant cell tumor
