# Supplementary material for: Cadherin-11 cooperates with inflammatory factors to promote the migration and invasion of fibroblast-like synoviocytes in pigmented villonodular synovitis
Source: Theranostics. 2020 Aug 21;10(23):10573–88. doi: 10.7150/thno.48666 (PMC7482803; doi:10.7150/thno.48666)
Supplement: Supplementary file 1 — Supplementary figures and tables. [file thnov10p10573s1.pdf]

Supplementary data

Cadherin-11 cooperates with inflammatory factors to promote the migration and invasion of fibroblast-like synoviocytes in pigmented villonodular synovitis

Chenxi Cao[1#], Fei Wu[1#], Xingyue Niu[1#], Xiaoqing Hu[1*], Jin Cheng[1], Yan Zhang[2], Chuanyun Li[3], Xiaoning Duan[1], Xin Fu[1], Jiying Zhang[1], Xin Zhang[1*], Yingfang Ao[1*]

1. Institute of Sports Medicine, Beijing Key Laboratory of Sports Injuries, Peking University Third Hospital, 49 North Garden Road, Haidian District, Beijing 100191, People's Republic of China
2. State Key Laboratory of Membrane Biology, Institute of Molecular Medicine, Peking University, 5 Yiheyuan Road, Haidian District, Beijing 100871, People's Republic of China
3. Institute of Molecular Medicine, Beijing Key Laboratory of Cardiometabolic Molecular Medicine, Peking University, 5 Yiheyuan Road, Haidian District, Beijing 100871, People's Republic of China

[#] Contributed equally to this work

[*]Corresponding authors: Yingfang Ao, E-mail: aoyingfang@163.com, Phone: 010-82267390, Fax: 86-10-62010440.

[*]Corresponding authors: Xin Zhang, E-mail: tozhangxin@sina.com, Phone: 010-82267390, Fax: 86-10-62010440.

[*]Corresponding authors: Xiaoqing Hu, E-mail: huxiaoqingbd01@sina.com, Phone: 010-82267390, Fax: 86-10-62010440.

Figure S1

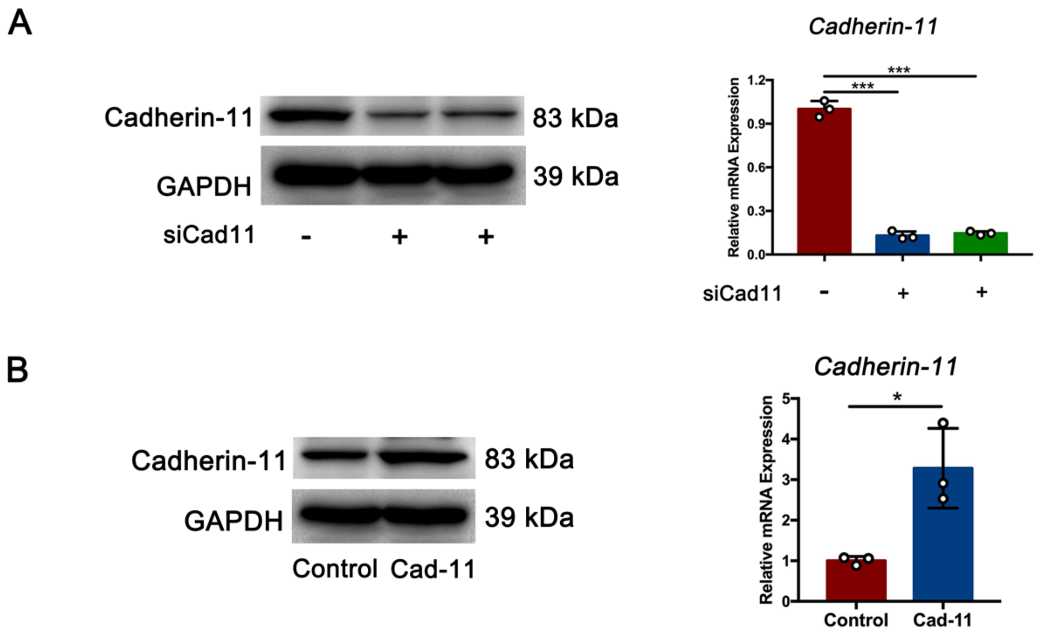

Figure S1. Validation of siCadherin-11 and overexpression plasmid in PVNS FLS.

(A) siCadherin-11 could knock down the expression of cadherin-11 at both the protein and mRNA levels in PVNS FLS.

(B) The cadhein-11 overexpression plasmid could up-regulate the expression of cadherin-11 at both the protein and mRNA level in PVNS FLS.

*$P < 0.05$; **$P < 0.01$; ***$P < 0.001$.

Figure S2

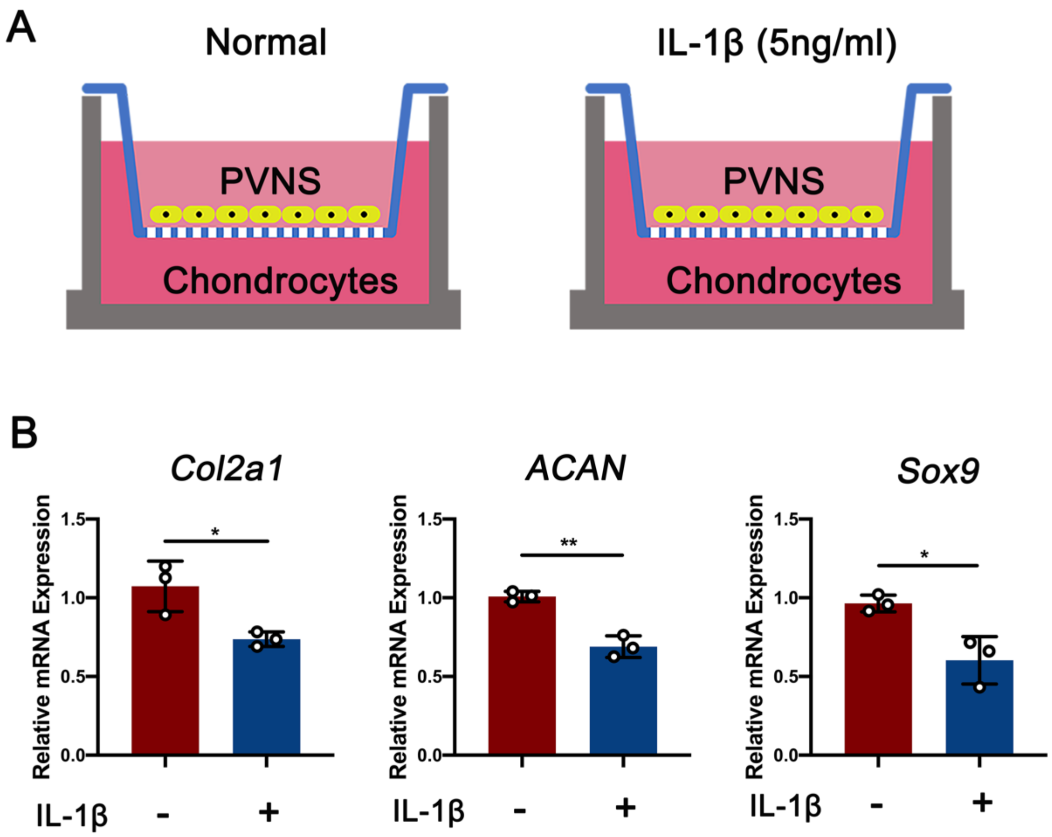

Figure S2. The co-culture analysis of chondrocytes and PVNS FLS activated by IL-1β.

(A) Schematic diagram of the co-culture system consisting of chondrocytes and PVNS FLS with or without IL-1β (5 ng/ml).

(B) The effects of IL-1β (5 ng/ml) on the mRNA expression of classic ECM-related genes in chondrocytes within the co-culture system after 72 h.

*$P < 0.05$; **$P < 0.01$; ***$P < 0.001$.

Figure S3

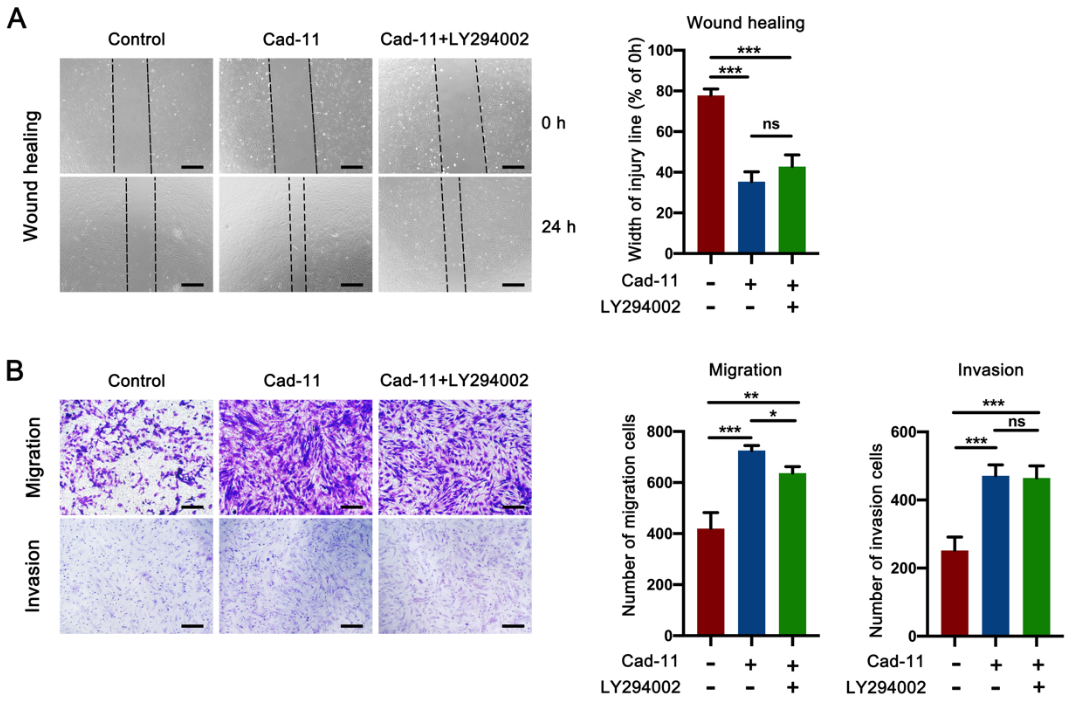

Figure S3. Inhibition of PI3K/Akt pathway could not decreased the migration and invasive capacity of PVNS FLS induced by cadherin-11 overexpression plasmid.

(A)Wound-healing assays were performed in PVNS FLS induced by cadherin-11 overexpression plasmid with or without the PI3K inhibitor, LY294002. Scale bar: 50 μm.

(B) Transwell assays were performed in PVNS FLS induced by cadherin-11 overexpression plasmid with or without the PI3K inhibitor, LY294002. Scale bar: 50 μm.

**P* < 0.05; ***P* < 0.01; ****P* < 0.001.
